# Perspectives on systematic review protocol registration: a survey amongst stakeholders in the clinical research publication process

**DOI:** 10.1186/s13643-023-02405-z

**Published:** 2023-12-14

**Authors:** Kim van der Braak, Pauline Heus, Claudia Orelio, Fredh Netterström-Wedin, Karen A. Robinson, Hans Lund, Lotty Hooft

**Affiliations:** 1 Cochrane Netherlands, Julius Center for Health Sciences and Primary Care, University Medical Centre Utrecht, Utrecht University, Utrecht, The Netherlands; 2 Research Support, Diakonessenhuis Utrecht, Bosboomstraat 1, Utrecht, 3582 KE The Netherlands; 3 School of Public Health and Community Medicine, University of Gothenburg, Gothenburg, Sweden; 4 Division of General Internal Medicine, Department of Medicine, Johns Hopkins University School of Medicine, Baltimore, MD USA; 5 Section for Evidence-Based Practice, Department of Health and Functioning, Western Norway University of Applied Sciences, Bergen, Norway

**Keywords:** Systematic review, Prospective protocol registration, Preregistration, Survey, Consolidated Framework for Implementation Research, Open science, Barriers, Facilitators

## Abstract

**Background:**

As systematic reviews (SRs) inform healthcare decisions, it is key that they address relevant questions and use rigorous methodology. Registration of SR protocols helps researchers identify relevant topics for future reviews and aims to prevent bias and duplication of effort. However, most SRs protocols are currently not registered, despite its significance. To guide future recommendations to enhance preregistration of SRs, it is important to gain a comprehensive understanding of the perspectives within the research community. Therefore, this study aims to examine the experiences with and factors of influence (barriers and facilitators) on prospective SR registration amongst researchers, peer reviewers and journal editors.

**Methods:**

Two different surveys were distributed to two groups: researchers and journal editors both identified from an existing sample of SRs. Researchers who indicated to have peer reviewed a SR were surveyed on their perspectives as peer reviewers as well. Survey design and analysis were informed by the Consolidated Framework for Implementation Research (CFIR). Shared and unique subthemes from the perspectives of researchers, peer reviewers and journal editors were identified and linked to the SR registration process (Innovation), to team, organisation (Inner setting) and (inter)national research community (Outer setting), and to characteristics of researchers, peer reviewers or journal editors (Individuals).

**Results:**

The survey’s response rates were 65/727 (9%) for researchers, of which 37 were peer reviewers, and 22/308 (7%) for journal editors. Most respondents (*n* = 76, 94%) were familiar with SR protocol registration and 81% of researchers had registered minimally one SR protocol. Shared SR registration process subthemes were the importance and advantages of SR protocol registration, as well as barriers such as a high administrative burden. Shared subthemes regarding the inner and outer setting centred on journal processes, external standards and time. Shared individual factors were knowledge, skills and awareness.

**Conclusions:**

The majority of the respondents were familiar with SR protocol registration and had a positive attitude towards it. This study identified suboptimal registration process, administrative burden and lack of mandatory SR protocol registration as barriers. By overcoming these barriers, SR protocol registration could contribute more effectively to the goals of open science.

**Systematic review registration:**

osf.io/gmv6z.

**Supplementary Information:**

The online version contains supplementary material available at 10.1186/s13643-023-02405-z.

## Introduction

Systematic reviews (SRs) synthesise evidence to inform clinical practice and thereby impact healthcare decision-making world-wide [1]. First, it is key that SRs answer clinically relevant questions by enquiring stakeholders. Secondly, it is important that SRs employ rigorous and transparent methods to provide valid results. Lastly, while SRs should be updated with the latest evidence, unintended replication of SRs should be avoided as it fails to contribute to new knowledge.

Prospective registration or publication of SR protocols was introduced with the aim to reduce publication and reporting bias, to prevent unplanned duplication, to facilitate review planning and updating, to optimise the utilisation of research funding and to create opportunities for methodological or collaborative research [2–4]. In line with clinical trial registration, SRs with protocol registration have been associated with higher methodological quality and better reporting than those without protocol registration [5–8]. SR protocols can be published in scientific journals like *BMJ Open* and *Systematic Reviews*. They can also be registered on digital platforms, either specifically for SRs, like the international prospective register of systematic reviews (PROSPERO), or on a general open science platform like the Open Science Framework (OSF). For Cochrane reviews, all protocols are published in the Cochrane Database of Systematic Reviews (CDSR) and automatically registered in PROSPERO.

Despite all these options, the percentage of SRs that have a registered protocol is still relatively low. Recent studies showed that 17–40% of SRs have a registered or published protocol [7, 9–12]. There are numerous examples of unplanned duplication [13, 14]. For example, regarding the treatment of stroke, 17 SRs including meta-analyses have been published, mostly within a timeframe of 6 months, using the results from the same three trials [13]. This excessive redundancy in SRs on a particular topic implies that scarce clinical research time and resources were wasted. This may have been prevented by prospective protocol registration.

In a recent survey, SR authors listed several reasons for failing to register SR protocols, including the lack of awareness, non-mandatory status, time-investment and fear of others stealing research ideas [15]. Further exploration of these reasons for non-registering is needed to understand which future actions will contribute to objectives of SR protocol registration. Since the publication process of SRs is heavily dependent on the editorial process, the effectiveness of SR protocol registration is also affected by the views and actions of journal editors and peer reviewers, which have not been explored yet. Previously, journal editors played a vital role in increasing trial registration after the introduction of mandatory clinical trial protocol registration by the International Committee of Medical Journal Editors (ICMJE) [16, 17]. Hence, journal editors may also play a vital role in shaping the future of SR protocol registration. Ultimately, this study aims to examine the experiences with and factors of influence (barriers and facilitators) for prospective SR protocol registration amongst researchers, peer reviewers and journal editors. By seeking further understanding of their experiences and perspectives using an implementation framework, we aim to provide the scientific community with recommendations that will contribute to the goals of open science.

## Methods

The study protocol was preregistered in the Open Science Framework (OSF) on July 8th, 2022 (osf.io/gmv6z) [18]. The writing of this manuscript is informed by the Checklist for Reporting of Survey Studies (CROSS) and the SUrvey Reporting GuidelinE (SURGE) [19, 20]. Related materials can be found on the OSF repository (https://doi.org/10.17605/OSF.IO/AT596).

### Consolidated Framework for Implementation Research (CFIR)

To address important aspects of implementation, which are essential for future actions, we used the CFIR to inform this research [21, 22]. The use of this framework allowed a structured assessment of barriers and facilitators. The framework provides an inventory of determinants over five domains that have been related to implementation and is applicable to a wide variety of settings. The first CFIR domain, ‘Innovation’, refers to the process and application of SR protocol registration or publication. Secondly, the ‘Inner setting’ and ‘Outer setting’ domains were combined and these represent the factors influencing SR protocol registration within a team, organisation or at a (inter)national level. Lastly, the ‘Individual’ domain represents characteristics of researchers, peer reviewers and journal editors that relate to SR protocol registration/publication. The CFIR domain, ‘Implementation process’, was not deemed applicable since this research did not assess any formal implementation strategies or activities.

### Surveys

Our team developed two surveys: one for researchers and another for journal editors. Researchers who also served as peer reviewers were provided with additional questions in their survey. The development of the survey started with a brainstorm session amongst four authors (PH, CO, LH and KvdB) to identify important topics. Then, a single author (KvdB) designed the survey questions and answering options based on the domains of the CFIR [21, 22] and barriers and facilitators for protocol registration, registered reports or preregistration identified in previous research [11, 15, 23–27]. The draft surveys were refined after discussion with the other three authors (LH, PH and CO). The survey was piloted on four junior researchers from our department to check completeness of the answering options, understanding of the questions and face validity. Both surveys included open questions, multiple choice, multiple answer and rating questions (e.g. range from 0 to 100 or Likert scales). Dependencies were used to ensure optimal survey flow, see Additional File 1. Out of 40 items for researchers, 25 items were deemed essential for the interpretation of our results and were therefore required to proceed with the survey. For journal editors, 28 out of 41 items were essential and required. The complete surveys can be found on OSF repository (https://doi.org/10.17605/OSF.IO/AT596).

The survey for researchers included a section of questions for peer reviewers. Researchers participating in the survey who indicated to have peer reviewed a systematic review were asked to complete this section from their perspective as peer reviewer. This survey included the following sections: (1) demographic information, (2) familiarity and experience with SR protocol registration or publication as a researcher, (3) potential barriers and facilitators as a researcher and (4) SR protocols during peer review (for those with peer reviewer experience only). Demographic data for researchers consisted of age, gender, country, years working in research, number of SRs co-authored and experience with SR protocol writing.

The survey for journal editors included three sections: (1) demographic information, (2) familiarity and editorial experience with SR protocol registration and (3) potential barriers and facilitators from an editorial perspective. For journal editors, the collected demographic data included age, gender, country, journal name (optional to ensure anonymity of journal editors), length of work experience and function specification (editor in chief/associate editor/other).

### Study sample and data collection

We used an existing sample of 357 SRs from a previous study to identify researchers who have published interventional SRs in the field of health(care) research and editors from journals in which these SRs were published [12]. These SRs were published in 2020 and 2021 and covered 63 different medical fields [12]. Email addresses from corresponding authors and/or first and last authors were collected from Web of Science (WoS). When no author email address could be retrieved through WoS, an additional manual internet search (Google search engine) was conducted to identify publicly available author email addresses. Email addresses of journal editors and editorial offices were retrieved from journal websites. High-ranking journals were not well represented in the sample of SRs (median impact factor of 3.8 and interquartile range from 2.6 to 5.4). Therefore, we invited journal editors from the ten highest ranked journals in WoS-category General and Internal Medical that publish SRs of health research to increase our chances that journal editors of high-ranking journals would be represented in our sample. Journal editors from Cochrane were not approached as (peer-reviewed) protocol registration is embedded in their distinct SR generation process.

All researchers received an email explaining the rationale of the study and were invited to participate in the study via a personal link to the survey. For journal editors with an identified personal email address, an email with a personal link to the survey was sent. If a general editorial email address was obtained, then an open link to the survey was sent. In those cases, the email included a request to forward the open link to all editorial staff of the journal. When proceeding to the survey via the link, participants were asked to provide informed consent. Participants who received a personal link to the survey and did not participate received four reminders (at 2, 4, 6 and 8 weeks) after the first email was send. We expected a response rate of 10%. To distribute the survey and collect the responses, we used an electronic data capture system (CastorEDC) secured by two-factor authentication [28] that allocated an ID number to each email address. The key to link the ID number to an email address was stored in a password secured environment separate from the responses. The survey for researchers and peer reviewers was distributed from July to October 2022 and for journal editors from October 2022 to January 2023. Participants received no incentives for participation. Collected email addresses for which no response was collected during the survey period were deleted from the system.

### Data analysis

Quantitative data from the survey were analysed descriptively and visually using R version 4.2.2 [29]. A survey response was deemed complete when all mandatory (essential) questions were filled out. Both complete and incomplete responses were used in the analysis. The number and frequencies were calculated for each of the multiple choice and ranking questions, using the number of respondents that answered the question as the denominator. Means with standard deviations and/or medians and interquartile ranges (IQR) were used to summarise continuous numeric data, depending on normality of the data (as determined by visual inspection). Open-ended questions were analysed using NVivo qualitative data analysis software following a thematic analysis approach [30]. Responses were first read and inductively coded per question by two researchers independently (KvdB, PH). Second, codes from both researchers were compared and final codes were determined through discussion by the two researchers. Then, all identified codes were deductively mapped onto the Innovation, Inner and outer setting and Individuals domains of the CFIR by a single researcher (KvdB) [21]. The inner and outer setting domains were combined since it depends on the respondent’s perspective what to call inner or outer setting. Final codes, presented as subthemes within each CFIR domain, were discussed with a second researcher (PH). Both qualitative, including relevant quotes, and quantitative data provided input for a narrative and visual synthesis of results.

## Results

### Response rate and characteristics of respondents

We invited 727 researchers and 308 journal editors to take part in the survey. In total, 66 of 727 researchers (50 complete and 16 incomplete responses), of whom 37 indicated to have peer reviewed a SR, provided informed consent. Twenty-three of 308 journal editors (16 complete and 7 incomplete responses) provided informed consent. One researcher and one journal editor who provided informed consent did not complete any of the survey questions, which leaves a sample size of 65 researchers (response rate of 9%), of which 37 were peer reviewers, and 22 journal editors (response rate 7%).

Characteristics of the respondents are shown in Tables 1 and 2. Most researchers were assistant, associate or full professors (*n* = 42, 64%) with more than 10 years of research experience (*n* = 37, 57%). Almost 40% of researchers had (co-)authored two to five SRs (*n* = 25) and another 42% had performed six or more SRs (*n* = 28). Like researchers, most journal editors with various editorial roles had more than 10 years of experience as editors (*n* = 9, 41%). A list of the journals represented by the editors is provided in Additional File 2. Of all article types accepted by these journals, the median percentage that were systematic reviews was 10% (IQR: 6 to 20%). Researchers and journal editors cover a wide range of clinical fields and researchers worked in diverse geographical locations (Additional File 3A).

**Table 1:** Characteristics of researchers (*n* = 65)

Characteristics of researchers	*N*	%
**Total no. researchers**	65	
**Gender**^**a**^		
Males	40	62%
Females	25	38%
**Age**^**a**^		
25–35	15	23%
36–45	27	42%
46–55	11	17%
56–65	10	15%
> 65	1	2%
I prefer not to say	1	2%
**Experience as a researcher**^**a**^		
< 2 years	1	2%
2–5 years	9	14%
6–10 years	18	28%
> 10 years	37	57%
**Academic position**^**a**^		
Bachelor or Master student	6	9%
PhD student	7	11%
Postdoc or assistant professor	15	23%
Associate professor	10	15%
Full professor	17	26%
Other	10	15%
**No. SRs (co-)authored**		
None	3	5%
1	9	14%
2–5	25	38%
6–10	10	15%
11–25	12	18%
> 25	6	9%
**Have you ever registered or published a SR protocol?** (***n*** = 59)		
Yes	48	81%
No, but I’m familiar with SR protocol registration or publication	8	13%
No, I’m not familiar with SR protocol registration or publication	3	5%

All questions above were mandatory. Of the 66 researchers that provided informed consent, one respondent did not fill out any of the questions and was therefore not represented in this table

*SR* Systematic review

^a^Respondents could indicate ‘I prefer not to say’

**Table 2:** Characteristics of journal editors (*n* = 22)

Characteristics of journal editors	*N*	%
**Total no. journal editors**	22	
**Editorial role**^**a**^		
Editor in chief	8	36%
Associate editor	5	23%
Executive editor	2	9%
Member of the editorial board	1	5%
Other	6	27%
I prefer not to say	0	0%
**Years working as journal editor**^**a**^		
< 2 years	1	5%
2–5 years	8	36%
6–10 years	4	18%
> 10 years	9	41%
**Familiarity with SR protocol registration or publication**		
Publication only	1	5%
Registration only	9	41%
Both registration and publication	10	45%
Not familiar with either	2	9%
**What percentage of accepted publications are systematic reviews or meta-analyses?**		
Median (IQR)	10% (6 to 20%)	
**Journal policy concerning SR protocol registration or publication** (***n*** = 17)		
Mandatory	4	24%
Recommended	8	47%
No journal policy	5	29%
**Indicated that journal follows ICMJE recommendations** (***n*** = 19)	18	95%
**What should the ICMJE recommend on SR protocol registration or publication?** (***n*** = 16)		
Current recommendations on data sharing adequately covers SR protocol registration	4	25%
More specific recommendations than in their current recommendations	3	19%
It should be required as a condition of consideration for publication	8	50%
Other	1	6%

All questions above were mandatory. Of the 23 journal editors that provided informed consent, one respondent did not fill out any of the questions and was therefore not represented in this table

*SR* Systematic review, *IQR* Interquartile range

^a^Respondents could indicate ‘I prefer not to say’

Most researchers and journal editors were familiar with SR protocol registration/publication (*n* = 76, 94%). Three researchers indicated that they had never co-authored a SR, and three researchers and two journal editors were not familiar with SR protocol registration/publication leading to the end of the survey for these respondents.

### CFIR domains

**Fig. 1:**
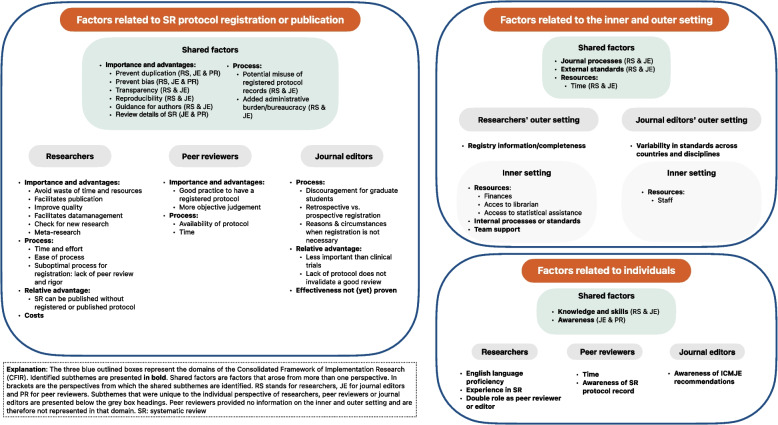
Visual presentation of the CFIR domains and identified subthemes

Identified subthemes (in italic) from the open-ended answers, supplemented with responses to closed-ended questions, are presented below according to the CFIR domains for the researchers’, peer reviewers’ and journal editors’ perspectives. Figure 1 presents a schematic overview of identified subthemes within each of these domains. Detailed and visual representation of responses to the closed-ended questions are presented in Additional File 3 and a full description of each subtheme and related quotes from each perspective are presented in Additional File 4.

#### Process and application of SR protocol registration/publication (Innovation domain)

Researchers, peer reviewers and journal editors provided rich data on characteristics and process of SR registration or publication. Identified subthemes were (1) *importance and advantages*, (2) *process*, (3) *relative advantage*, (4) *costs* and (5) *effectiveness* (Fig. 1).

All three groups highlighted the *importance and advantages* of SR protocol registration/publication, such as prevention of duplication, reproducibility, transparency and prevention of bias. This was confirmed with ranking questions on intended outcomes, see Additional File 3B. SR protocol registration/publication also had specific advantages for each group. For researchers, developing and then registering or publishing the protocol guided them through the SR process and facilitated publication. For journal editors and peer reviewers, an available SR protocol helped them evaluate submitted SRs in more detail.

Quotes related to *importance and advantages* subtheme:

‘*It is important to have a clear protocol for conducting systematic review to prevent bias. Registering systematic review prevents duplication of work by others.*’—researcher

‘*The number of SR submissions has exploded. We want to publish novel studies that advance clinical practice, and avoid publishing SRs that have already been recently done. We need a way to track what has already been done.*’—journal editor

‘*I consult the protocol as a peer-reviewer especially to check if there [are] any *ad hoc* decisions not reported in the manuscript and judge the risk of selective reporting. In any of these cases, authors must clearly justify these decisions in the manuscript for transparency purposes.*’—peer reviewer

Although the importance and advantages were stressed, researchers and journal editors also reported barriers and limitations of SR protocol registration/publication in the subthemes: *process* and *relative advantage*. With regard to the *process*, both journal editors and researchers mentioned the increased bureaucracy, added administrative burden and potential misuse of protocol records. Researchers gave a median score of 50 (IQR: 20 to 80 on a scale from 0—no impact to 100—full impact on the decision to register or publish a SR protocol) for the impact of potential for stealing research ideas on their decision to register/publish a SR protocol. Researchers expressed varying views of the ease of the processes (see quotes below), even regarding the same registry such as PROSPERO, which was most frequently used. In general, registration in PROSPERO or OSF was regarded as easy (41% and 61%) or very easy (23% and 25%). On the other hand, protocol publication in a journal or the CDSR was deemed to be more complex (Additional File 3C). Journal editors conveyed contrasting opinions about prospective and retrospective protocol registration. Several journal editor statements implied that for some reasons and circumstances registration/publication of a SR protocol may not be necessary. However, these reasons and circumstances were not further specified. Additional File 3D lists more advantages and disadvantages given by journal editors. Barriers for peer reviewers concerning the process were the lack of public availability of SR protocol records and the additional time needed to assess the protocol.

Quotes illustrating different views regarding the process of protocol registration or publication:

‘*I have found PROSPERO registration to be very slow and thus have not pursued it after the first couple of attempts to register 2 different protocols.*’—researcher

‘*PROSPERO is an easy and fast website that publishes SR protocols. On the other hand, publishing SR protocols in formally [scientific] journals sometimes can take long time. This situation can delay the writing and the final SR development…*’—researcher.

The *relative advantage* subtheme was illustrated by description of researchers balancing their time and effort, either by using faster registries as alternatives to avoid delays or by not registering at all. This shows that some researchers may decide to not register/publish SR protocols, since it is not required for publishing the SR. Journal editors argued that SRs without a protocol could still be of good quality and that protocol registration may be less important for SRs compared to clinical trials.

The *effectiveness* subtheme was highlighted by a single journal editor who was not convinced that SR protocol registration had proven itself as he/she was faced with several duplicate SR submitted. A multiple-choice question about effectiveness in the researchers’ survey showed that one-third of the researchers believed that the evidence supporting the benefits for SR protocol registration was adequate. The remaining researchers were unsure about the evidence (*n* = 21, 44%) or found the evidence not adequate (*n* = 11, 23%).

Only researchers reported on the subtheme *costs* related to SR protocol registration/publication. As SR protocol registration/publication may take some additional time, it may subsequently also bring additional costs.

‘*Unfortunately, the main resource is economic, to pay for the expenses of publications, and not to depend so much on work at the University to have time to dedicate to the development of research protocols.*’—researcher.

#### Factors within a team, organisational or at a (inter)national level (Inner and outer setting domain)

Researchers and journal editors shared three subthemes: (1) *journal processes*, (2) *external standards* and (3) *time as a resource* (Fig. 1). There were no subthemes identified from the peer reviewer’s perspective.

Researchers referred to* journal processes* as being dependent on journal requirements, while journal editors referred to it as their internal processes within the editorial team. Researchers reported barriers such as a lack of journals or unclear conditions for accepting SR protocols for publication and expressed a need for copyright or embargo times imbedded into journal processes. Facilitating factors for researchers to register/publish SR protocols were journal policies (*n* = 36, 78%) and having better chances for publishing (*n* = 27, 59%); more factors of influence are shown in Additional File 3E. Journal editors proposed various ways or described attempts to deal with unregistered SRs, such as post hoc registration, rejection of manuscript or demanding to explain or acknowledge the lack of a protocol record. Half of the journal editors believed SR protocol registration/publication should be a requirement for publication (*n* = 8, 50%, Table 2). More detailed characteristics of journal processes regarding SR protocol publication are summarised in Additional File 3F.

Quotes illustrating the subtheme *journal processes*:

‘*[there is a need for] copyright!! Please protect the contents from scooping, at least for a limited time (6 months/a year). You can create collaboration with the journals and convince the editors to check your list during the preliminary assessment of manuscripts.*’—researcher

‘*We encountered a case that completed the peer review process and accepted without registration. We discussed it at the editors meeting and decided to ask the author to register. However, it was not possible to save the data after extraction. Therefore, we decided to publish it with summarizing this process.*’—journal editor

Both researchers’ and journal editors’ use of SR protocol registration/publication was influenced by *external standards* and *time as a resource*. Researchers referred to the PRISMA reporting guidelines, the Joanna Briggs Institute (JBI) and Cochrane as being external standards and journal editors referenced the ICMJE guidance.

Several subthemes unique to researchers’ inner and outer setting were identified. One being the *registry information/completeness*. Researchers depended on available registry information to identify which topics were already in the process of review and one respondent pointed out that PROSPERO records are often not updated. Other factors influencing SR protocol registration/publication amongst researchers were *internal processes or standards* at team or institute level, *finances, librarian, and statistical assistance as a resource* and *team support*. Researchers rated their time for registering or publishing SR protocols more adequate (median 60, IQR: 28 to 83) than their finances (median 20, IQR: 4 to 71) on a scale from 0 (not adequate) to 100 (fully adequate).

Unique subthemes identified amongst journal editors were (1) *variability in standards* and (2) *journal editorial staff as a resource*. Journal editors reported a *variability in standards* for SR methodology and protocol registration/publication and differences in interpretations of the term ‘systematic review’ between various research fields and countries. This was a barrier for them to integrate structured or standardised requirements for SR protocol registration/publication. It was therefore argued by some journal editors that they needed flexibility in standards for SR protocol registration/publication. The lack of *journal editorial staff as a resource* was perceived as a potential barrier for journal editors to evaluate SR protocols during the journal editorial process.

#### Characteristics of researchers, peer reviewers and journal editors related to SR protocol registration/publication (Individual domain)

**Fig. 2:**
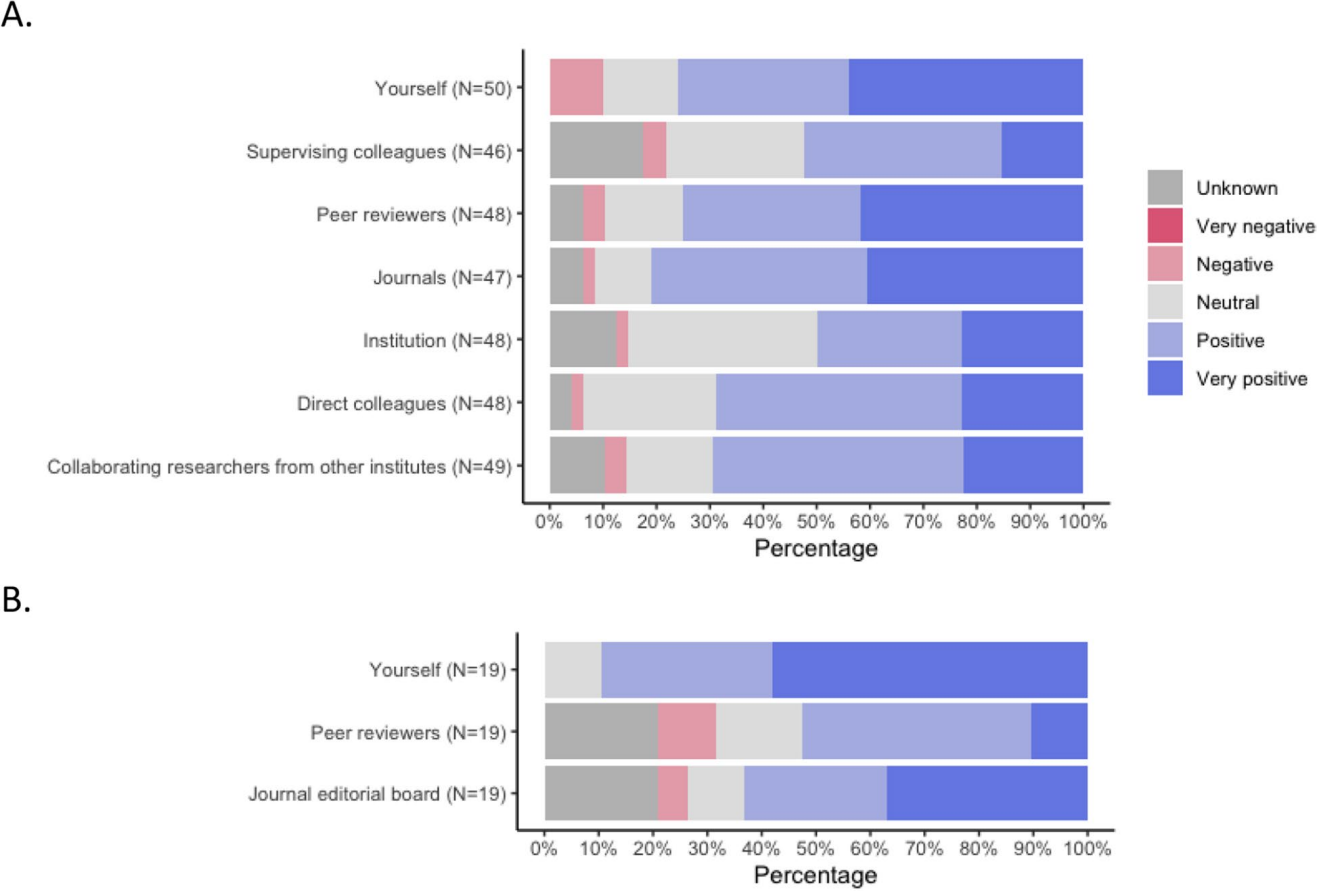
Attitudes of **A** researchers and **B** journal editors towards SR protocol registration or publication Participants were asked to score the attitude of themselves and others regarding protocol registration/publication

Both surveys contained closed-ended questions on the attitude towards SR protocol registration/publication. Researchers and peer reviewers were also asked about their SR protocol registration/publication behaviour. The attitude of both researchers (including peer reviewers) and journal editors towards SR protocol registration/publication was positive (*n* = 16 researchers, 32%; *n* = 6 journal editors, 32%) or very positive (*n* = 22 researchers 44%; *n* = 11 journal editors, 58%), see Fig. 2.

From the researchers who registered/published a protocol for at least one SR (*n* = 48, 81%), most had registered/published a protocol for 80 to 100% of SRs (*n* = 24, 50%), followed by those who had registered/published a protocol for 20% or less of SRs (*n* = 14, 29%) (Additional File 3G). The remaining 21% (*n* = 10) of researchers reported to register a protocol for 20 to 79% of their SRs. Researchers’ behaviour regarding consulting SR protocol registries or records are portrayed in Additional File 3H.

Individual factors that may affect SR protocol registration/publication identified from open-ended questions were coded in the following subthemes: *knowledge and skills* (researchers and journal editors), *awareness* (journal editors and peer reviewers), *experience with SRs* (researchers), *double role as peer reviewer or editor* (researchers), *English language proficiency* (researcher), *time* (peer reviewers) (Fig. 1).

Quotes related to individual factors:

‘*It [decision to publish or register the SR protocol] depends on the time available and especially on the knowledge of those writing the protocol.*’—researcher

‘*I don’t have a lot of systematic review studies*’—researcher

‘*I am unaware of specific guidance [of the ICMJE] on review protocol registration*’—journal editor

‘*I consulted [SR protocols as peer reviewer] when I have the time*’—peer reviewer

Researchers gave the adequacy of their knowledge of SR protocol registration/publication a median rating of 90 (IQR: 80 to 100) on a scale from 0 (not adequate) to 100 (fully adequate). Journal editors indicated that time and resources of themselves affected them negatively (*n* = 5, 33%), as well as the time and resources of peer reviewers (*n* = 6, 40%, Additional File 3I).

Most researchers learned about SR protocol registration/publication through reporting guidelines (*n* = 33, 65%, Additional File 3J.1). Most journal editors learned about SR protocol registration/publication through journal articles (*n* = 13, 65%, Additional File 3J.2).

Peer reviewers indicated that they were usually not (*n* = 9, 24%) or never (*n* = 11, 30%) stimulated by journal editorial staff to consult SR protocol records in the peer review process (Additional File 3K). However, half of the peer reviewers indicated to consult SR protocols always (*n* = 9, 24%) or most of the time (*n* = 11, 30%) during peer review, when available (Additional File 3K).

## Discussion

This study provides important insights into the experiences of researchers, peer reviewers and journal editors regarding SR protocol registration and publication. Guided by the CFIR, we identified shared and unique subthemes from the perspectives of researchers, peer reviewers and journal editors. These subthemes related to the SR registration process (Innovation domain), factors within a team, organisation or (inter)national research community (Inner and outer setting domain) and individual factors (Individuals domain). Our findings shed light on the multifaceted nature of SR protocol registration and provide a comprehensive understanding of the topic.

In general, researchers, including the subset of peer reviewers, and journal editors expressed a positive attitude towards SR protocol registration and publication. They agreed upon the importance of the intended outcomes associated with prospective SR protocol registration/publication such as avoiding unnecessary duplication and reducing publication and selective reporting bias. However, the respondents also highlighted certain negative aspects of SR registration, which were in line with a previous study [15]. These negative aspects included increased bureaucracy, additional time and resources needed, and concerns regarding the potential of others stealing research ideas. In current times of high pressure to publish with limited time and resources available, these negative aspects may outweigh the benefits in some cases.

Contrary to a previous study that identified a lack of knowledge or awareness amongst researchers as a main barrier to SR protocol registration [15], our study did not find supporting evidence for this claim. In fact, only 6% of the respondents were not familiar with SR protocol registration or publication. This suggests that there may have been an improvement in the knowledge and awareness of SR protocol registration/publication over time.

We identified two other main categories of barriers: (1) different platforms and processes for SR protocol registration/publication and (2) lack of consensus on mandatory requirements and standards. The first category concerns the different platforms and processes for SR registration/publication. Each method of SR protocol registration, including publishing a SR protocol in a journal, has its own advantages and disadvantages. Some individuals may favour protocols published in a journal because the feedback received during the peer-review process can lead to more rigorous SR methods. Additionally, published protocols may also have greater visibility than registered records, which was highlighted by another study [11]. However, the publication and peer review process can be time-consuming, resulting in delaying the SR. In our survey, researchers also indicated the limited number of journals accepting SR protocols and uncertainty regarding which SR protocols may be of interest to journals to publish (e.g. those employing novel methods or pertaining to a certain topic). To overcome these barriers, respondents expressed the need for faster publication processes for SR protocols and acceptation of SR protocols for publication by all journals that accept SRs for publication. Compared to publication in a journal, SR protocol registration was found to be easy and fast, but current SR protocol platforms lack opportunities for other researchers to provide feedback. To enhance the number of rigorous protocol records, it may be beneficial for platforms such as PROSPERO to consider implementing open peer review, similar to the F1000 platform [31]. This would enable a more transparent and collaborative process in assessing protocol quality. Research registration platforms could also include other features that support users, such as assistance in searching relevant records or reminders to similar records before submission of a SR protocol [32]. Another potential barrier is the lack of an overview of all SRs protocol records, as the number of databases for SR protocols may increase over time. One solution could be the development of a search interface that combines all sources for SR protocols, like the International Clinical Trials Registry Platform (ICTRP) curated by the World Health Organization (WHO) [33], or incorporation of review protocols in Epistemonikos (a search interface for systematic reviews) [34]. Such a search interface would facilitate easier access to SR protocols, enabling researchers, peer reviewers and journal editors to evaluate the need for a review topic more effectively and prevent redundant reviews to be published.

A second group of barriers relates to a lack of consensus on mandatory requirements and standards for SR protocol registration/publication. Our results show that there is no consensus amongst researchers and journal editors on whether SR protocol registration/publication should be required for publication of a SR. Respondents referred to SR registration/publication as good practice but did not always voice opinions that SR protocol registration/publication should be mandatory. Also, some argued that mandatory SR protocol registration/publication may be more important for some types of SRs; however, which types of SRs this statement referred to remained unclear. The latter has also been highlighted in the survey study of Rombey et al. [11]. Even if SR protocol registration/publication would be required for publication, there are conflicting opinions on allowing retrospective registration, and for which type of reviews this needs to be applied to. Journal editors raised concerns about the different standards for SR protocol registration/publication between different research fields and different interpretations of a ‘systematic review’, which partly prevented them from implementing mandatory SR protocol registration/publication. To overcome these barriers, journals should clearly state their journal requirements and preferences for SR protocol registration/publication. The ICMJE could play a vital role in formulating these specific requirements and preferences, following the example of clinical trial registration [16]. Their involvement would also reduce variety in requirements and standards between journals and provide guidance for researchers conducting SRs.

### Strengths, limitations and generalisability

We incorporated several actions to promote our response rate, including optimising the survey length, ensuring data security and privacy and sending multiple reminders at different days and times. Despite these, our response rates were just below 10%, which was similar to a previous survey study amongst SR authors on SR protocol registration or publication [15]. Another survey, which targeted SR authors through PROSPERO records and likely included participants more familiar with the topic, achieved a higher response rate (34%) [11]. This variation may be indicative of either non-response bias or the varying levels of interest in the topic amongst the surveyed population.

Despite the limited sample size overall, sufficiently rich data from the open questions allowed us to explore experiences and opinions of different perspectives using qualitative thematic analysis. The CFIR enabled us to systematically assess potential factors of influence (barriers and facilitators) for successful implementation, resulting in more rigorous methodology. To limit the length of the survey, the number of questions on the peer review perspective was relatively low compared to the other two perspectives. Therefore, the perspective of peer reviewers may not be as saturated compared to the researchers and journal editors. Our findings may not fully represent the perspectives of researchers or journal editors who are unfamiliar or lack experience with SR protocol registration/publication or with SRs in general, since they may have been less likely to respond to our survey. Our study included researchers and journal editors from a diverse sample of interventional SRs published in 2020 and 2021 from a wide range of medical fields and countries. While our survey questions were not focused on a specific type of SRs, the applicability to non-interventional SRs, such as diagnostic test accuracy or prognosis reviews, may be limited.

## Conclusions

This survey study showed that researchers, peer reviewers and journal editors were familiar with and generally positive about SR protocol registration/publication. However, identified barriers related mainly to the suboptimal process of SR protocol registration/publication. These barriers need to be addressed, so that SR protocol registration or publication can contribute more effectively to the goals of open science. For instance, there is a need for faster processes for publishing SR protocols in journals. Additionally, providing options for (open) peer reviewing registered protocols could enhance the quality and rigor of SRs (protocols). Since there is no consensus on mandatory SR protocol registration/publication, journals should clearly communicate their requirements and preferences in their instructions to authors. More specific requirements and standards on SR protocol registration/publication provided by the ICMJE may encourage adherences to good practices in the field.

### Supplementary Information

**Additional file 1.** Schematic flow of questions and sections of the survey for (A) researchers and peer reviewers and (B) journal editors.

**Additional file 2.** List of journals.

**Additional file 3.** Responses to multiple choice and rating questions.

**Additional file 4.** Detailed information of CFIR domains and identified subthemes from each perspective.

**Additional file 5.** Checklist for Reporting Of Survey Studies (CROSS).

## Data Availability

The datasets and scripts for analysis of the current study are available in the OSF repository: https://doi.org/10.17605/OSF.IO/AT596 [18].

## Abbreviations

CDSR: Cochrane Database of Systematic Reviews
CFIR: Consolidated Framework for Implementation Research
CROSS: Checklist for Reporting of Survey Studies
ICMJE: International Committee of Medical Journal Editors
ICTRP: International Clinical Trials Registry Platform
IQR: Interquartile range
JBI: Joanna Briggs Institute
PRISMA: Preferred Reporting Items for Systematic Reviews and Meta-Analyses
PROSPERO: International prospective register of systematic reviews
SR: Systematic review
SURGE: SUrvey Reporting GuidelinE
OSF: Open Science Framework
WHO: World Health Organization
WoS: Web of Science
