# Supplementary material for: Perspectives on systematic review protocol registration: a survey amongst stakeholders in the clinical research publication process
Source: Syst Rev. 2023 Dec 14;12:234. doi: 10.1186/s13643-023-02405-z (PMC10720136; doi:10.1186/s13643-023-02405-z)
Supplement: Supplementary file 2 — Additional file 2. List of journals. [file 13643_2023_2405_MOESM2_ESM.pdf]

Additional file 2. List of journals

1. Brain Injury
2. Cardiovascular Revascularization Medicine
3. Developmental Medicine and Child Neurology
4. Endocrinology, Diabetes and Metabolism
5. European Journal of Clinical Pharmacology
6. European Journal of Orthodontics
7. Evaluation & the Health Professions
8. International Journal of Nursing Studies
9. JBI Evidence Synthesis
10. Journal of Geriatric Physical Therapy
11. Journal of Palliative Medicine- Palliative Medicine Reports
12. Journal of the American Pharmacists Association
13. Nutrition Today- Food Chem Toxicology- Nutrients- Toxicology Research and Application
14. Pediatrics
