# Supplementary material for: Perspectives on systematic review protocol registration: a survey amongst stakeholders in the clinical research publication process
Source: Syst Rev. 2023 Dec 14;12:234. doi: 10.1186/s13643-023-02405-z (PMC10720136; doi:10.1186/s13643-023-02405-z)

Additional file 3. Responses to multiple choice and rating questions

A. Detailed characteristics on researchers and journal editors

1. Research topics of researchers and journal editors based on Web of Science categories. Respondents could indicate multiple topics.

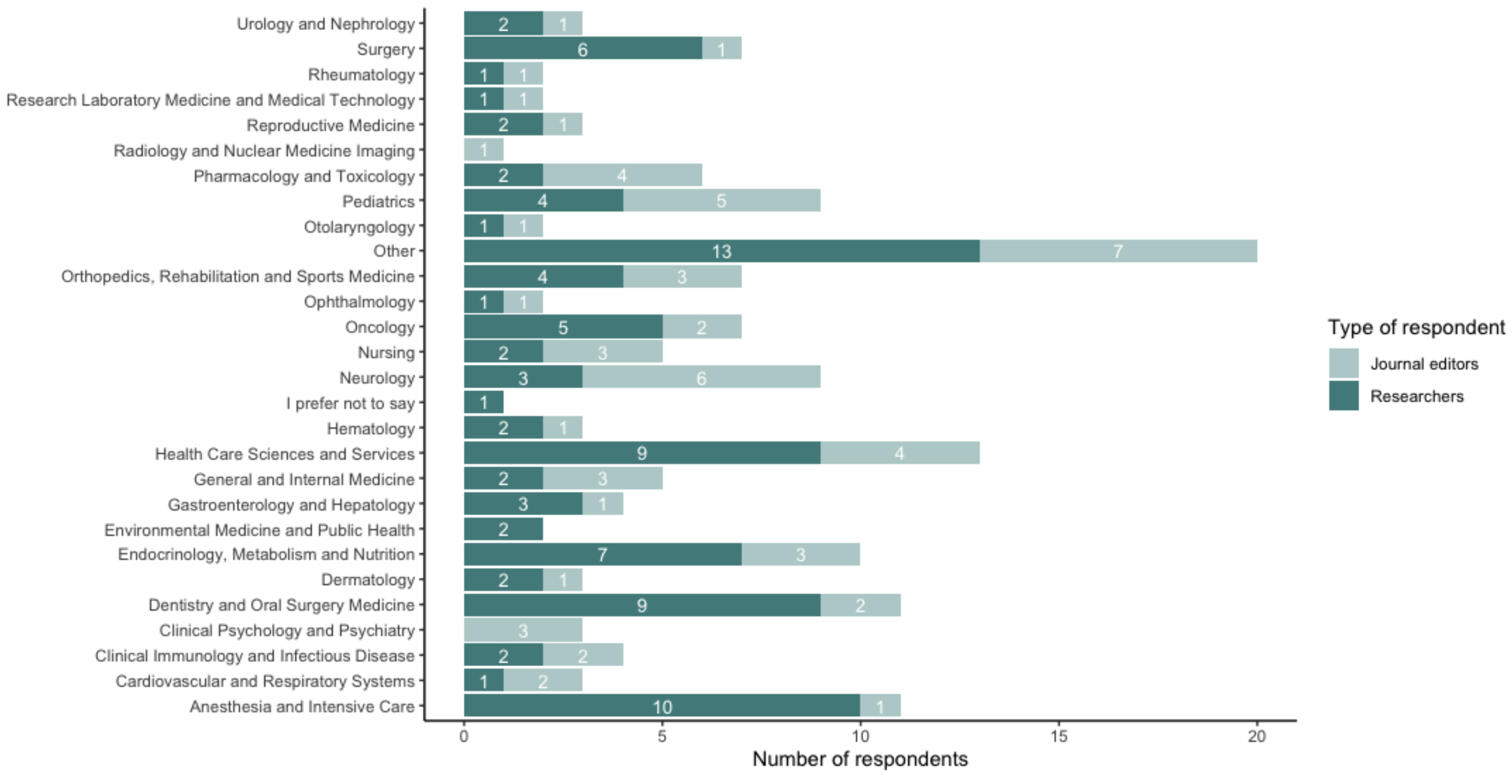

2. Country of origin of researchers

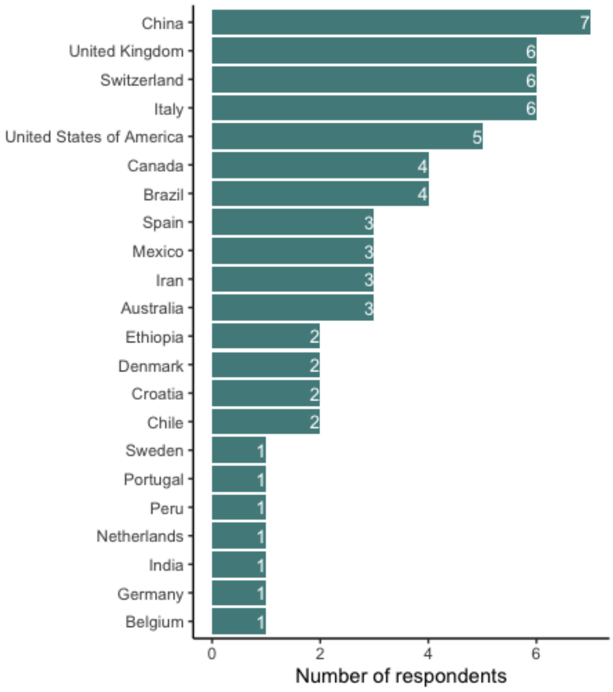

B. Importance and capability ratings

1. Ratings for importance and capability of SR protocol registration/publication to achieve the intended outcomes as listed by PROSPERO by journal editors and researchers.

**Intended outcomes**	**Researchers**		**Journal editors**
	**Rating for importance*** Median (IQR)	**Rating for capability**** Median (IQR)	**Rating for capability***** Median (IQR)
Avoid unnecessary duplication	100 (80 to 100)	80 (50 to 100)	80 (70 to 90)
Reduce publication and selective reporting bias	90 (80 to 100)	80 (50 to 90)	70 (50 to 80)
Assist in planning and updating of reviews	80 (50 to 100)	80 (60 to 90)	80 (80 to 85)
Help make efficient use of research funding and reduce waste	80 (50 to 100)	70 (40 to 80)	50 (40 to 85)
Create opportunities for methodological and other collaborative research	80 (50 to 95)	50 (30 to 80)	50 (30 to 60)

Respondents provided a score from 0 to 100 for each of the listed intended outcomes. Researchers indicated how capable SR protocol registration was to achieve the outcome and how important this outcome was to them. Journal editors only scored capability on this scale and rated the importance on a Likert scale shown in Additional Figure 3B2. IQR: Interquartile range.
*n=44 for outcome help make efficient use of research funding and reduce waste, n=45 researchers for all other outcomes, **n=46,***n=13 journal editors

2. Importance of intended outcomes of SR protocol registration or publication, rated by journal editors (n=14).

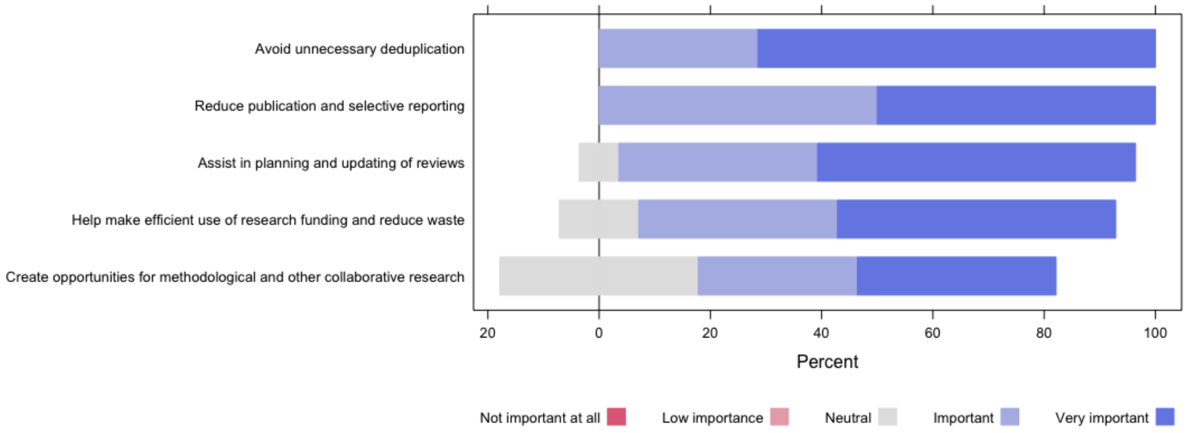

C. Methods to register or publish a SR protocol

1. Researchers indicating which methods they have used and how complicated these methods were.

Method for SR registration or publication	Researchers						
	% (N) that have used this method[1]		How easy do researchers find the following methods for SR protocol registration/publication[2]:				
			Very easy	Easy	Neutral	Somewhat complex	Very complex
**CDSR**	20% (8)		0%	38%	12%	25%	25%
**PROSPERO**	96% (46)		23%	41%	32%	5%	0%
**OSF**	18% (8)		25%	62%	0%	0%	12%
**Other registry**	10% (4)		0%	67%	0%	33%	0%
**Publication of protocol**	66% (29)		4%	26%	26%	44%	0%

[1] total number of responses of researchers varied per listed method from 41 to 48, therefore the N and percentages differ for each method.
[2] Values are percentage of the number of researchers who have indicated that they have used the corresponding method.
CDSR: Cochrane Database of Systematic Reviews, PROSPERO: International Prospective Register of Systematic Reviews, OSF: Open Science Framework

2. Journal editors indicating which methods for SR protocol registration/publication they encounter most often.

Method for SR registration or publication[1]	Journal editors (N=16)				
	How often do journal editors encounter the following methods for SR protocol registration/publication:				
	Very often	Often	Sometimes	Almost never	Never
**PROSPERO**	44%	19%	19%	19%	0%
**OSF**	6%	0%	25%	50%	19%
**Other registry**	0%	6%	31%	38%	25%
**Publication of protocol**	0%	13%	50%	25%	13%

[1] Journal editors were not asked about the Cochrane Database of Systematic Reviews, as the editors were from other journals than Cochrane.
PROSPERO: International Prospective Register of Systematic Reviews, OSF: Open Science Framework

D. Advantages and disadvantages for journal editors

Advantages (1) and disadvantages (2) of SR protocol registration for journal editors (n=15).

1

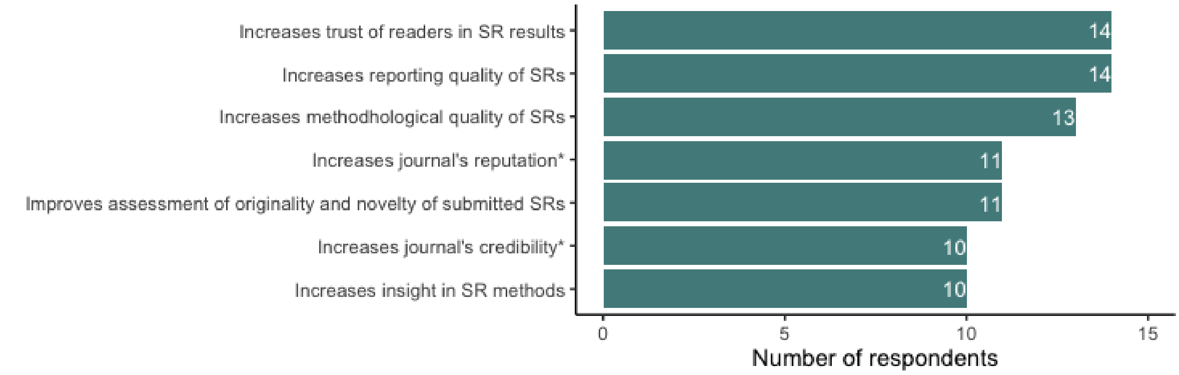

2

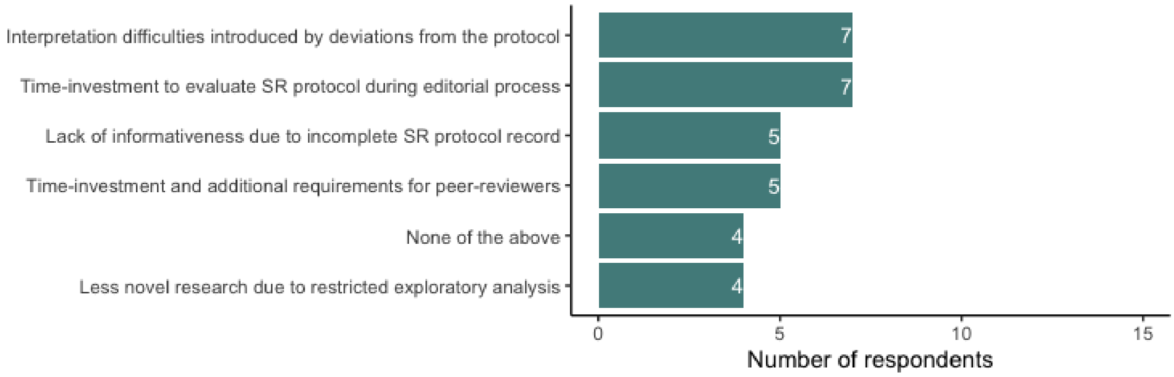

Journal editors could indicate one or more advantages or disadvantages listed. None of the journal editors selected the following response options for the advantages of SR protocol registration:

- Can be a quality feature that could justify a raise in fees for memberships*
- Can be a quality feature that could justify a raise in the publication charges*
- None of the above

*if SR protocol registration/publication would be a criterium for publishing a SR.

E. Factors stimulating researchers to register/publish SR protocols
Researchers indicated which kind of recognition or rewards they get when they register/publish their SR protocol (1) and which policies stimulate SR protocol registration (2). Researchers could indicate more than one answer. (n=46).

1

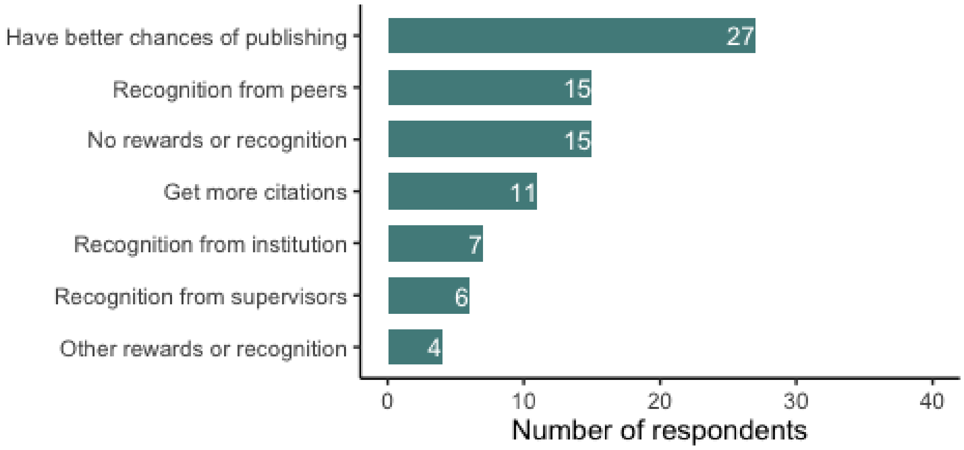

2

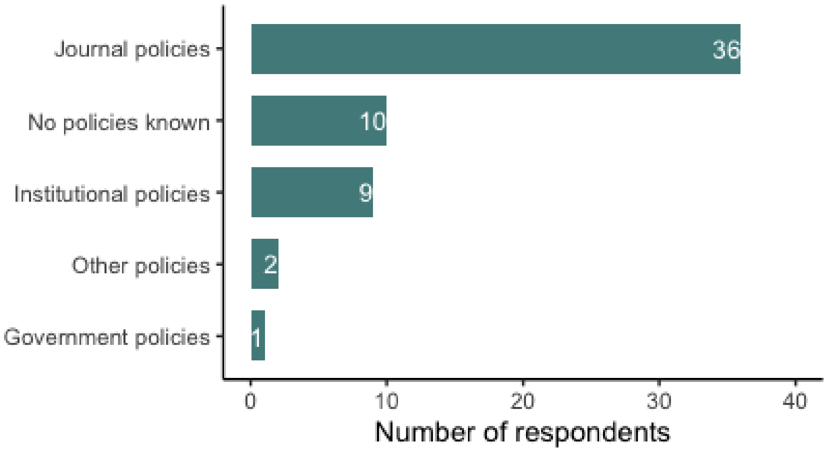

F. Journal process characteristics

1. Rating by journal editors for how often the situations listed in the table, pertaining to SR protocol registration, occurred from 0 (Never) to 100 (Always).

How often on a scale from 0 to 100:	Median (IQR)	N
does your journal accept protocols for SRs for publication?	1 (0 to 9)	20
does your journal accept submitted SRs that have a protocol registered or published?	32.5 (10 to 80)	18
does your journal recommend authors to remove text or sections on protocol registration	0 (0 to 1)	17
does your journal recommend authors to include text or sections on protocol registration	51 (1 to 100)	17
do you, in your role as editor, check protocol registries to see whether a submitted SR is original or novel?	25 (1 to 100)	17
do you, in your role as editor, check publications on SR topics to see whether a submitted SR is original or novel?	75 (10 to 100)	17

SR: Systematic reviews, IQR: Interquartile range

2. Journal communication methods regarding SR protocol registration/publication policy (n=12 journal editors). Journal editors could indicate multiple options.

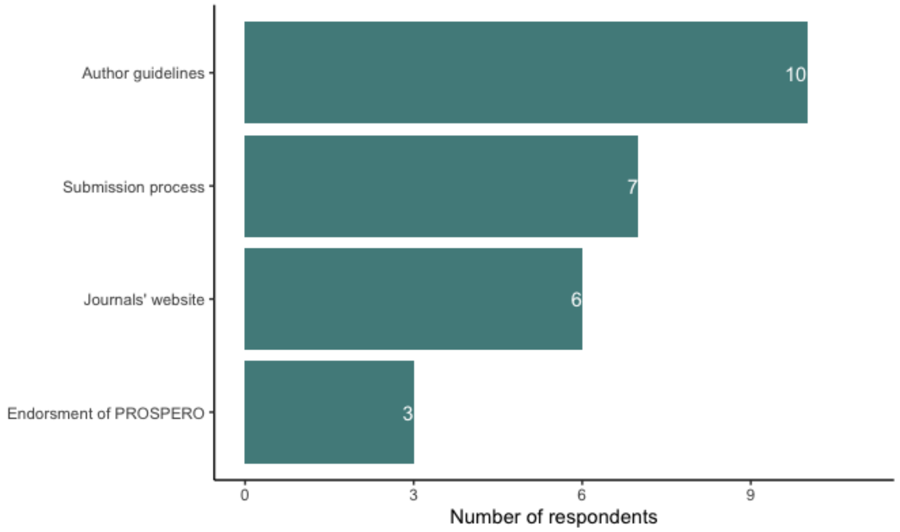

G. Registration behaviour of researchers

Percentage of systematic reviews for which researchers have registered or published a protocol (n=48)

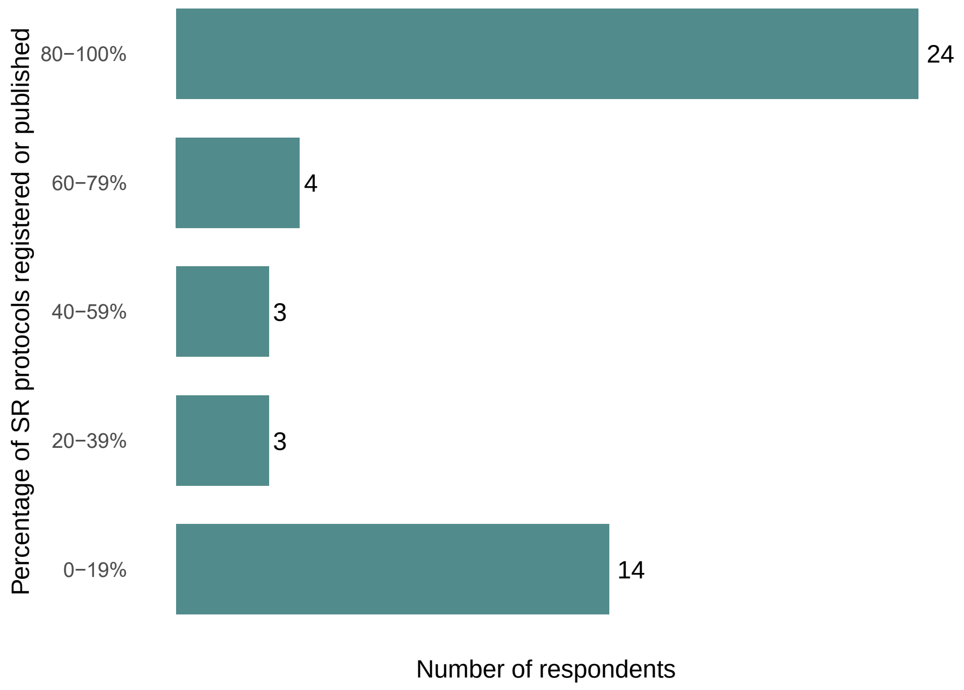

H. Situations and frequencies in which researchers consult SR protocols

**Situation:**		How **often** have you **consulted SR protocols** in each of these situations;						**N**
		Never	*Usually not*	*Some-times*	*Most of the time*	*Always*	*NA*	
Before conducting a new SR	N	3	0	5	15	32	0	55
	%	5,5%	0,0%	9,1%	27,3%	58,2%	0,0%	
Before conducting a new trial	N	2	5	9	10	17	10	53
	%	3,8%	9,4%	17,0%	18,9%	32,1%	18,9%	
Before conducting other types of research	N	4	11	14	9	11	4	53
	%	7,5%	20,8%	26,4%	17,0%	20,8%	7,5%	
To check methodological quality of a published SR	N	4	10	14	11	13	0	52
	%	7,7%	19,2%	26,9%	21,2%	25,0%	0,0%	

I. Factors influencing SR protocol registration use among journal editors during the editorial process

Journal editors rated the positive or negative impact (1) and the importance (2) of each of the listed factors on their editorial decisions (n=15)

1

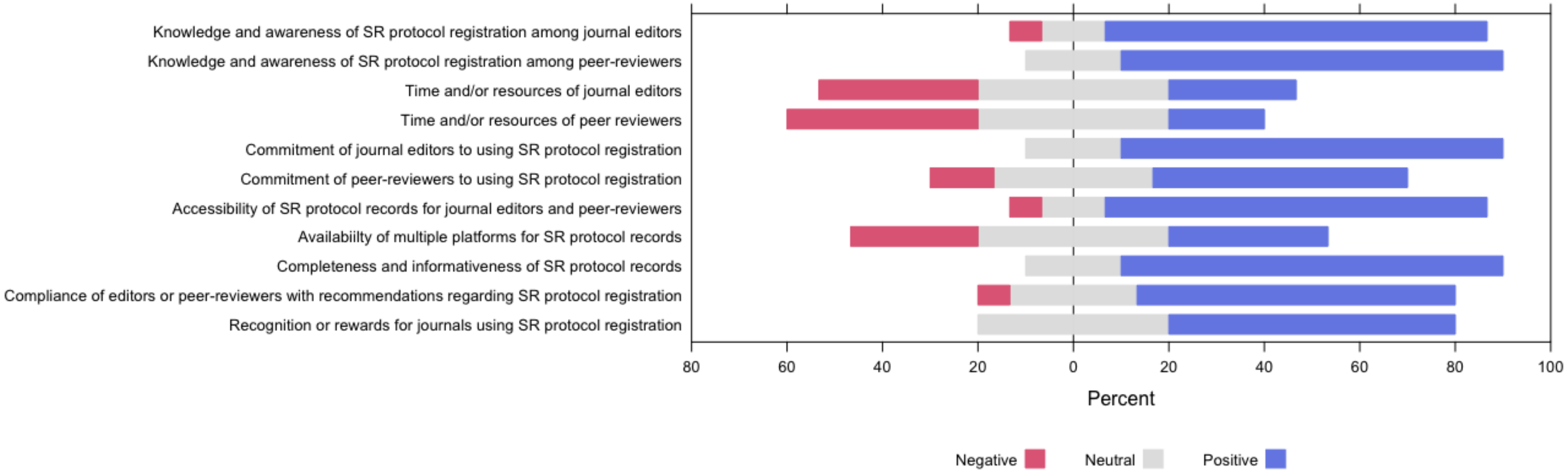

2

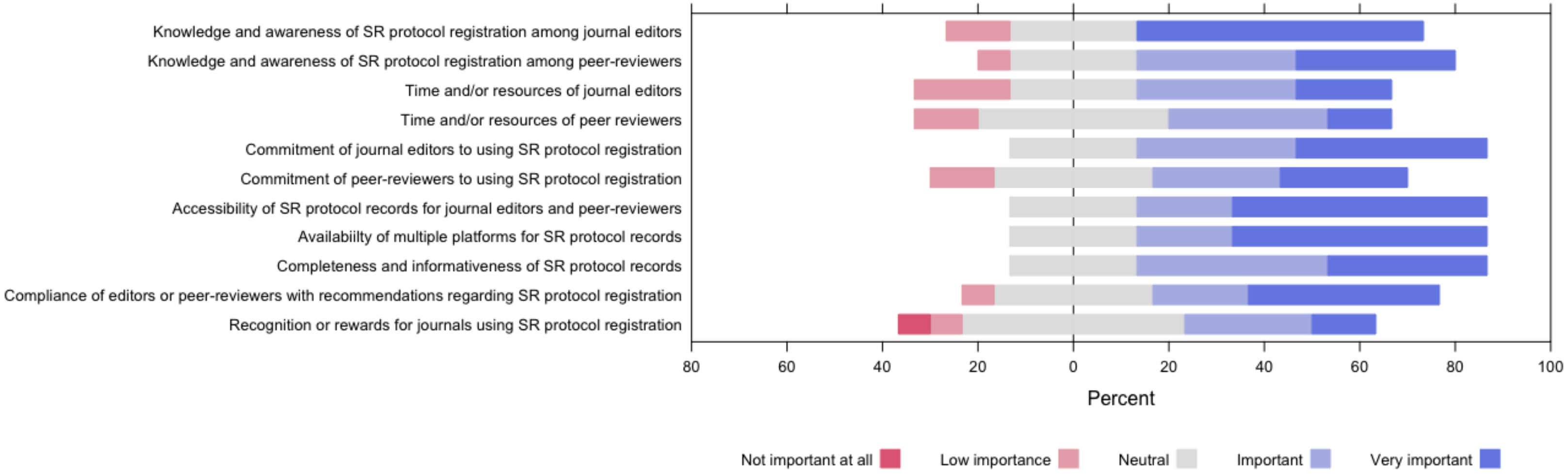

J. Knowledge of SR protocol registration or publication

Through which means did 1) researchers (n=51) and 2) journal editors (n=20) learn about SR protocol registration or publication.

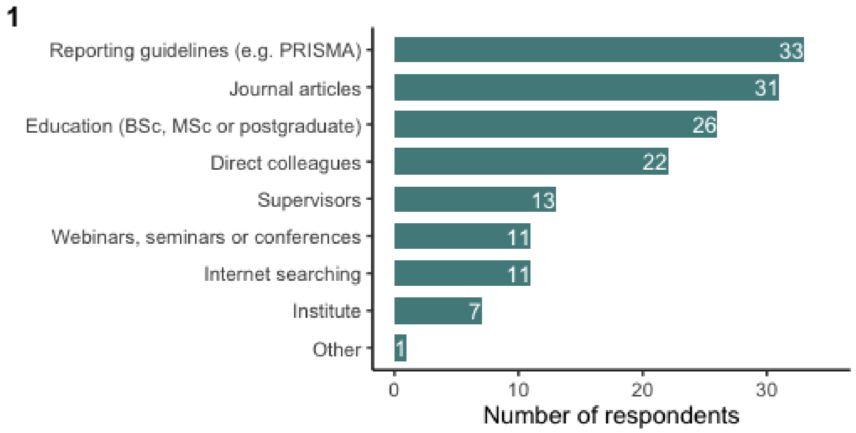

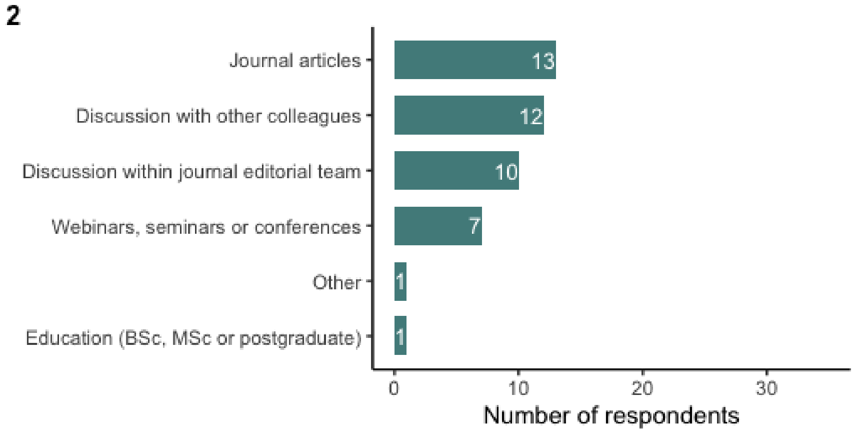

K. Questions to peer-reviewers

Researchers indicating that they have peer-reviewed systematic reviews were considered peer reviewers (n=37) and were asked the following questions on peer review.

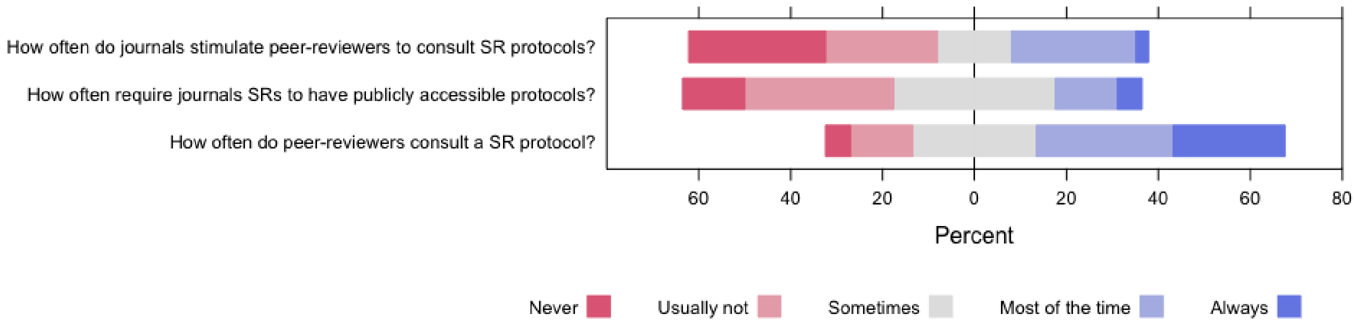

Supplement: Supplementary file 3 — Additional file 3. Responses to multiple choice and rating questions. [file 13643_2023_2405_MOESM3_ESM.pdf]
