# Supplementary material for: Perspectives on systematic review protocol registration: a survey amongst stakeholders in the clinical research publication process
Source: Syst Rev. 2023 Dec 14;12:234. doi: 10.1186/s13643-023-02405-z (PMC10720136; doi:10.1186/s13643-023-02405-z)
Supplement: Supplementary file 4 — Additional file 4. Detailed information of CFIR domains and identified subthemes from each perspective. [file 13643_2023_2405_MOESM4_ESM.pdf]

Additional file 4. Detailed information of CFIR domains and identified subthemes from each perspective

A. Researchers

**Domain & subthemes**	**Explanation and meaning of theme**	**Quotes**
**Innovation domain**		
Importance & advantages	Researchers indicated that SR protocol registration or publication was important for:<br>• Prevention of duplication<br>• Reproducibility<br>• Transparency<br>• Avoidance of waste of time and resources<br>• Prevention of (publication and reporting) bias<br><br>Researchers also mentioned that having a registered/published protocol had some advantages:<br>• Improves quality of the SR<br>• Facilitates publication<br>• Assists authors in planning the SR and provides guidance through the SR process<br>• Registration through a platform such as OSF could facilitate data-management<br><br>The availability of SR protocol records through a registry or publication allowed researchers to check for new research or do meta-research.	"It is important to have a clear protocol for conducting systematic review to prevent bias. Registering systematic review prevents duplication of work by others."<br>"Because we believe it is as essential as it is for a clinical trial to prevent publication bias and reporting bias."<br>"It is a good practice, to foster transparency and reduce bias."<br>"Registering protocol helps us to have a plan for the research and makes it easy to conduct the SR."<br>"For OSF (not PROSPERO), we are able to log the progress of the paper within the system, and very useful for me to upload there additional supplements that I don't want to add to the submission to the journal or that there are too long for journal supplement (e.g., excel files)"<br>"I study research methodology so I study their content for various research questions."
Process	Registration and publication of SR protocol records required additional time and effort of researchers which lead to a longer time needed before the final SR was published. Some researchers indicated differences in various registries or publication in terms of time-investment needed, delays, ease and format of the process. PROSPERO was indicated to be fast and easy by one researcher, but very slow by another. Another researcher critiqued the lack of peer review resulting in less rigor, insinuating that if protocol records need to be peer-reviewed and rigorous to be valuable.<br>There was one researcher who described their experience of potential misuse of their registered SR protocol, resulting in not being able to publish their review. This experience also led to this researcher no longer being willing to register (or publish) any SR protocol.<br><br>The decision where to register is not straightforward: there are many options to take, each with their own limitations and strengths as illustrated by the last quote.	"Sometimes we prepared the protocol and then we need to complete the information in PROSPERO that is double work."<br>"We usually go for PROSPERO, but due to delay we have now been using more the OSF, as an alternative or as complement..."<br>"I have found PROSPERO registration to be very slow and thus have not pursued it after the first couple of attempts to register 2 different protocols."<br>"PROSPERO is an easy and fast website that publishes SR protocols. On the other hand, publishing SR protocols in formally [scientific] journals sometimes can take long time. This situation can delay the writing and the final SR development…"<br>"PROSPERO publishes protocols that are not peer reviewed and lack rigurosity."<br>"We registered a systematic review protocol and made a submission to a journal. But the peer review process took about 6 months, and in the mean time, the editor informed us that a similar article had already been published. The sad thing about the publication is that the published article was submitted long after we submitted our manuscript. We reported the case to the ethical review board of both journals but with no effect."<br>"When we decided to publish is because it is a systematic or scoping review quite complex and one author has the time to invest in the publication process. Whereas in the case of the

		registration is when we do not have enough time. Another option is to publish in a repository or not a peer review journal."
Relative advantage	Some researchers indicated that SRs could be published without having a registered or published protocol. Besides it not being a mandatory practice, one researcher even argued that it was not favoured or appreciated during the publication process. Weighing of importance & benefits against the disadvantages is called relative advantage.	"Since the protocol registration is not a mandatory, appreciated, or favored practice during the publication process, it sounds unimportant."<br>"We failed … to publish protocols multiple times because of vague and imprecise reasons while the SR finally gets published in high impact journals (IF > 5)."<br>"admin burden without benefit"
Costs	Time of researchers equals costs. The time researchers spend on preparing a protocol for registration or publication, can't be spend on other projects.<br>Publication of SR protocols also introduces direct costs in the form of publication charges.	"Unfortunately, the main resource is economic, to pay for the expenses of publications, and not to depend so much on work at the University to have time to dedicate to the development of research protocols."
**Outer setting domain**		
Journal processes	Researchers depended on journal processes to being able to publish their SR or SR protocols. One of the researchers indicated a lack of journals who accept SR protocols. Another quote suggests that journals are not clear about which SR protocols are eligible for publication. Several researchers mentioned the need for protection of stealing research ideas through copy right or embargo periods, which would need to be imbedded into journals processes, to be successful.	"Copy right!! Please protect the contents from scooping, at least for a limited time (6 months/a year). You can create collaboration with the journals and convince the editors to check your list during the preliminary assessment of manuscripts."<br>"We failed … to publish protocols multiple times because of vague and imprecise reasons"<br>"[need for] a journal that publishes [SR] protocols"<br>"[need for] faster review/publishing time"
External standards	Researchers indicated several standards and requirements for SR protocol registration from journals (including Cochrane and JBI) and organisations. Reporting guidelines and other templates and standardized protocols could also contribute to the awareness of, and knowledge needed for SR protocol registration or publication.	"clinical practice guidelines consider only registered SR"<br>"We are also still using PROSPERO because some journal require specifically this database"<br>"Protocols for Cochrane reviews are always registered"<br>"[need for] Better templates and standardized protocols for systematic reviews of different kinds"
Registry information/completeness	SR protocol records are often consulted by researchers before writing/conducting their own SR (protocol). The status of these records needs to be up to date, so that researchers can correctly identify which SR topics have (not) been done.	"Authors register SRs there as ongoing and often they discontinued the SR and did not update the systems, which discourages other from pursuing that topic as they think someone is already doing that topic"
**Inner setting domain**		
Resources	Researchers depend on the resources available to them within their team or within their institution/organisation. Some indicated that SR protocol registration or publication would be facilitated if they had more time, finances, or (more) access to a librarian and/or a statistician.	"[need for] Full time statistician for the beginning. Part-time librarian would help, especially when having a PhD/ master student."<br>"[need for] time and finances"
Internal process at team or department level	Several researchers indicated that SR protocol registration was part of internal processes in their team or institute.	"standard procedure is to register before take off."<br>"It is a requirement in our process to make sure we all know what we are going to do and that it is feasible and relevant."
Team support	The decision to register or publish the protocol of a SR was not made by a single researcher, but usually through several researchers involved. Researchers mainly described support from the author team.	"Usually, this decision is up to the principal author, but all team strongly suggests to register protocol."
**Individual domain**		
Knowledge and skills	Researchers need adequate knowledge of and skills for protocol registration/publication when preparing the protocol.	"It [decision to publish or register the SR protocol] depends on the time available and especially on the knowledge of those writing the protocol."

English language proficiency	Common registries for SR protocol registration are in English. Researchers would need a certain proficiency in the English language to be able to register a SR protocol there.	"The information need to be submitted in English and not all researchers in Peru know the language."
Experience with SR	Some researchers only occasionally conduct a SR. Researchers less experienced in SRs may be less likely to register/publish a protocol due to a lack of knowledge, skills or awareness.	"I don't have a lot of systematic review studies"
Double role as peer reviewer or editor	Some researchers indicated that they have also acted as a peer reviewed and/or as a journal editor, and used SR protocol records in that role as well.	"[I consult SR protocol registries] when acting as peer reviewer and/or Editor to scientific journals."

B. *Journal editors*

**Domain & subthemes**	**Explanation and meaning of theme**	**Quote**
**Innovation domain**		
Importance & advantages	Journal editors highlighted that SR protocol registration or publication was important for:<br>• Prevention of duplication<br>• Reproducibility<br>• Transparency<br>• Increased credibility and trust<br><br>SR protocol records could help journal editors in the evaluation of submitted SRs and could assist SR authors to develop a proper design.	"Important for transparency, evaluation and replication"<br>"I would tend to agree that in most cases, systematic reviews that have registered protocols would be more trustworthy than those that do not have registered protocols."<br>"The number of SR submissions has exploded. We want to publish novel studies that advance clinical practice, and avoid publishing SRs that have already been recently done. We need a way to track what has already been done."<br>"[SR protocol registration/publication] urges the authors to develop a proper design"
Process	Several process factors were mentioned by journal editors. Journal editors acknowledged that SR protocol registration/publication would add to complexity and bureaucracy, especially when requiring it for publication. One journal editor referred to the potential misuse of SR protocol records within the competitive academic environment to publish first.<br>Some journal editors mentioned that for certain types of SRs registration would not be needed. However, it remained unclear for which SRs this applied to.<br><br>One journal editor highlighted that PROSPERO instructions discouraged SR authors to register their protocol. This journal editor elaborated on several submitted SRs for which no protocols were registered in PROSPERO because they were done as part of graduate thesis.	"May decrease the willingness to perform SRs due to increased bureaucracy"<br>"Prospective protocol registration may be used by others to generate an own systematic review faster than those who registered their protocol and have it published first."<br>"PROSPERO does not accept registration of "done as part of training courses, modules or other 'mini' reviews" … I've spoken with several graduate students who interpreted that as preventing them from registering their SR that was done as part of their thesis/dissertation."<br>"They should be able to register retrospectively IF their study is unique/novel/hasn't been done in recent years."<br>"Retrospective registration is a nonsense and should be discouraged."<br>"I also think there should be flexibility because there are circumstances in which I could see valid reasons why a systematic review would not have a registered protocol."
Relative advantage	Some journal editors who recognized the importance and benefits of SR protocol registration/publication, indicated that SRs without a registered protocol could still be valuable or that protocol registration was less necessary for SRs compared to trials.	"While protocols can be a tool to help improve standard there are downsides and lack of a protocol does not invalidate a good review."<br>"I do not feel this as mandatory as in case of clinical trials, but as strongly recommended"

Effectiveness	One journal editor highlighted that current use of protocol registration/publication in SRs, has not led to less duplicate SRs.	"I see potential, but judging by the number of systematic reviews we receive that often overlap, I don't see this potential being realized."
**Outer setting domain**		
External standards	Journal editors and editorial processes were guided by recommendations and guidance provided by the International Committee of Medical Journal Editors (ICMJE).	"As a new Editor these [ICMJE recommendations] are extremely helpful"
Variability in standards across countries and disciplines	Journal editors depend to a certain extent on submitted SRs from a certain field and variety in submitted publications made structured or standardized requirements for SR protocols difficult. In some fields, contexts or places, SR protocol registration is not well-known or practiced. There are also different interpretations of the term 'systematic review'.	"Some very high quality reviews emanate from places/contexts where the 'standard' of advance protocol specification is not widely adopted. This applies to cross disciplinary research and publications from some countries."<br>"There are competing issues in relation to diverse reviews and the widespread use of the terminology 'systematic review' that make it hard to standardise the requirements for advanced specification of protocols and registration."
**Inner setting domain**		
Journal processes	Journal editors make decisions about SRs within the context of the journal editorial team they work with. Within some journals the processes that involve SR protocol registration were part of routine, whilst in other cases there was discussion about how to deal with (un)registered SRs. Journal editors also described desirable processes that were seemingly not part of their current practice. Conflicting opinions were stated on whether retrospective registration should be allowed or encouraged during editorial processes.	"We encountered a case that completed the peer review process and accepted without registration. We discussed it at the editors meeting and decided to ask the author to register. However, it was not possible to save the data after extraction. Therefore, we decided to publish it with summarising this process."<br>"we do maintain flexibility related to prior registration of human subjects studies. This is primarily because we published a lot of practice-related research and per-registration is still not a common occurrence. We do routinely ask authors to register their studies (post-hoc registration) during the revision process if a paper moves to that stage."<br>"Defer the ms [manuscript/SR authors] to another journal [if the SR has no protocol registered or published]. [We] must educate investigators interested in SR to register the study and follow SR protocols."<br>"[journal editors need to] require authors to acknowledge lack of registration as a limitation."<br>"I think journals that require protocol registration should spell out why they require it and allow authors to explain if there are reasons they need to deviate from the standardized protocol in a particular systematic review."
Resources	One journal editor indicated that the time and staff needed are disadvantages or barriers for journal editors to integrate SR protocol evaluation within the journal processes.	"Time and staff bandwidth are key factors"
**Individuals domain**		
Knowledge and skills	Journal editors not only mentioned the need for their own skills and knowledge, but also referred to the knowledge and skills of SR authors.	"Training new editors and authors [would facilitate SR protocol registration]"
Awareness of ICMJE recommendations	One journal editor mentioned that his/her lack of awareness of the ICMJE recommendations on SR protocol registration indicated that he/she may not follow all the recommendations given. Although this journal editor was unaware of the specific guidance by the ICMJE, the journal did follow the ICMJE guidance in general and SR protocol registration was recommended by the journal. Being aware of guidance is a prerequisite to follow specific guidance, but journal editors may also be aware of SR protocol registration in general through other sources of information.	"I am unaware of specific guidance [of the ICMJE] on review protocol registration"

C. *Peer reviewers*

Domain & subthemes	Explanation and meaning of theme	Quote
**Innovation domain**		
Importance & advantages	SR protocol seemed to be seen as a helpful source of detailed information during the peer review process. It facilitated reviewing the SR methodology in general, bias and duplication. An available SR protocol was considered good practice and allowed for a more objective peer review judgement.	"It seems appropriate to review the protocols because I can order the ideas, comments and observations that I have in doubt and that I want to confirm with the evidence that the review of the protocols gives me."<br>"[I consult SR protocol records during peer review] to check if there is another research group working on the same idea."<br>"I consult the protocol as a peer-reviewer especially to check if there is any ad hoc decisions not reported in the manuscript and judge the risk of selective reporting. In any of these cases, authors must clearly justify these decisions in the manuscript for transparency purposes."<br>"Having a pre-registered protocol is part of the recognised methodology of SRs."<br>"[I consult SR protocol records during peer review] to be more objective in my judgement, to check the methodology"
Process	A potential barrier for using SR protocols during peer review is the lack of public availability of SR protocols.<br>Evaluating the SR protocol during peer review, may also require additional time of peer reviewers.	"Often the review protocol is not preregistered, or is mentioned as being stored in some academic department of the university and not publicly accessible."<br>"Time constraints"
**Individuals domain**		
Awareness during peer review	Peer reviewers need to be aware of SR protocol records during peer review for them to use them during evaluation. Additional instructions or having the protocol record readily available to them may be	"Don't always think of it [consulting protocols during peer review]."<br>"Would be useful if journal would include with mss [manuscript] a link to protocol (if not already included in mss [manuscript]."
Time	Peer reviewers may have limited time to perform peer review or have other priorities which allows less time to perform peer review and evaluating SR protocol records.	"I consulted [SR protocols as peer reviewer] when I have the time"
