# Supplementary material for: Perspectives on systematic review protocol registration: a survey amongst stakeholders in the clinical research publication process
Source: Syst Rev. 2023 Dec 14;12:234. doi: 10.1186/s13643-023-02405-z (PMC10720136; doi:10.1186/s13643-023-02405-z)
Supplement: Supplementary file 5 — Additional file 5. Checklist for Reporting Of Survey Studies (CROSS). [file 13643_2023_2405_MOESM5_ESM.docx]

Additional file 5. Checklist for Reporting Of Survey Studies (CROSS)

| **Section/topic** | **Item** | **Item description** | **Reported where** |
| --- | --- | --- | --- |
| **Title and abstract** | | |  |
| Title and abstract | 1a | State the word “survey” along with a commonly used term in title or abstract to introduce the study’s design. | The word “survey” is mentioned in the title and in the abstract. |
|  | 1b | Provide an informative summary in the abstract, covering background, objectives, methods, findings/results, interpretation/discussion, and conclusions. | Abstract covers background, objectives, methods, results and conclusion. Also includes preregistration in OSF. |
| **Introduction** | | |  |
| Background | 2 | Provide a background about the rationale of study, what has been previously done, and why this survey is needed. | See introduction |
| Purpose/aim | 3 | Identify specific purposes, aims, goals, or objectives of the study. | See end of introduction on page #3 |
| **Methods** | | |  |
| Study design | 4 | Specify the study design in the methods section with a commonly used term (e.g., cross-sectional or longitudinal). | See methods section: Study sample and data collection. Respondents could provide a single response. Study collected qualitative and quantitative data. |
|  | 5a | Describe the questionnaire (e.g., number of sections, number of questions, number and names of instruments used). | See methods section: Surveys. Full surveys are available through OSF repository. |
| Data collection methods | 5b | Describe all questionnaire instruments that were used in the survey to measure particular concepts. Report target population, reported validity and reliability information, scoring/classification procedure, and reference links (if any). | See methods section: Surveys. Self-developed survey, with rationale reported in methods section: Surveys. Full surveys are available through OSF repository. |
|  | 5c | Provide information on pretesting of the questionnaire, if performed (in the article or in an online supplement). Report the method of pretesting, number of times questionnaire was pre-tested, number and demographics of participants used for pretesting, and the level of similarity of demographics between pre-testing participants and sample population. | See methods section: Surveys. Pilot conducted on junior researchers from our department. |
|  | 5d | Questionnaire if possible, should be fully provided (in the article, or as appendices or as an online supplement). | Full surveys are available through OSF repository. |
| Sample characteristics | 6a | Describe the study population (i.e., background, locations, eligibility criteria for participant inclusion in survey, exclusion criteria). | See methods section: Study sample and data collection for eligibility criteria. See first paragraph of results section and Tables 1 and 2 |
|  | 6b | Describe the sampling techniques used (e.g., single stage or multistage sampling, simple random sampling, stratified sampling, cluster sampling, convenience sampling). Specify the locations of sample participants whenever clustered sampling was applied. | See methods section: Study sample and data collection. |
|  | 6c | Provide information on sample size, along with details of sample size calculation. | Part of the protocol registered and available through OSF repository. Definitive sample size reported in the first paragraph of results section. |
|  | 6d | Describe how representative the sample is of the study population (or target population if possible), particularly for population-based surveys. | See first paragraph of results section and Tables 1 and 2 |
| Survey  administration | 7a | Provide information on modes of questionnaire administration, including the type and number of contacts, the location where the survey was conducted (e.g., outpatient room or by use of online tools, such as SurveyMonkey). | See methods section: Study sample and data collection. |
|  | 7b | Provide information of survey’s time frame, such as periods of recruitment, exposure, and follow-up days. | See methods section: Study sample and data collection. |
|  | 7c | Provide information on the entry process:  –>For non-web-based surveys, provide approaches to minimize human error in data entry.  –>For web-based surveys, provide approaches to prevent “multiple participation” of participants. | See methods section: Study sample and data collection. Where possible, personal links were used preventing multiple participation. |
| Study preparation | 8 | Describe any preparation process before conducting the survey (e.g., interviewers’ training process, advertising the survey). | Preparation of the survey and the study is part of the protocol available through OSF repository. |
| Ethical considerations | 9a | Provide information on ethical approval for the survey if obtained, including informed consent, institutional review board [IRB] approval, Helsinki declaration, and good clinical practice [GCP] declaration (as appropriate). | See section: Ethics approval, consent to participate and privacy, at the end of the manuscript. |
|  | 9b | Provide information about survey anonymity and confidentiality and describe what mechanisms were used to protect unauthorized access. | See section Ethics approval, consent to participate and privacy, at the end of the manuscript. |
| Statistical  analysis | 10a | Describe statistical methods and analytical approach. Report the statistical software that was used for data analysis. | See methods section: Data analysis. |
|  | 10b | Report any modification of variables used in the analysis, along with reference (if available). | Not applicable. |
|  | 10c | Report details about how missing data was handled. Include rate of missing items, missing data mechanism (i.e., missing completely at random [MCAR], missing at random [MAR] or missing not at random [MNAR]) and methods used to deal with missing data (e.g., multiple imputation). | See methods section: Data analysis. |
|  | 10d | State how non-response error was addressed. | Repeated reminders see methods section: Study sample and data collection. Since it is a descriptive study, non-response error may only limit the generalizability of the results. |
|  | 10e | For longitudinal surveys, state how loss to follow-up was addressed. | Not applicable. |
|  | 10f | Indicate whether any methods such as weighting of items or propensity scores have been used to adjust for non-representativeness of the sample. | Not applicable. |
|  | 10g | Describe any sensitivity analysis conducted. | Not applicable. |
| **Results** | | |  |
| Respondent characteristics | 11a | Report numbers of individuals at each stage of the study. Consider using a flow diagram, if possible. | See first paragraph of results section and Tables 1 and 2. |
|  | 11b | Provide reasons for non-participation at each stage, if possible. | Reasons for non-participation are acknowledged in general in the discussion section on strengths and limitations. |
|  | 11c | Report response rate, present the definition of response rate or the formula used to calculate response rate. | See first paragraph of results section and Tables 1 and 2. |
|  | 11d | Provide information to define how unique visitors are determined. Report number of unique visitors along with relevant proportions (e.g., view proportion, participation proportion, completion proportion). | Not applicable: participants had a personal link to the survey. |
| Descriptive  results | 12 | Provide characteristics of study participants, as well as information on potential confounders and assessed outcomes. | See first paragraph of results section and Tables 1 and 2. Aim of survey was descriptive, so confounding was not applicable. |
| Main findings | 13a | Give unadjusted estimates and, if applicable, confounder-adjusted estimates along with 95% confidence intervals and p-values. | Not applicable. |
|  | 13b | For multivariable analysis, provide information on the model building process, model fit statistics, and model assumptions (as appropriate). | Not applicable. |
|  | 13c | Provide details about any sensitivity analysis performed. If there are considerable amount of missing data, report sensitivity analyses comparing the results of complete cases with that of the imputed dataset (if possible). | Not applicable. |
| **Discussion** | | |  |
| Limitations | 14 | Discuss the limitations of the study, considering sources of potential biases and imprecisions, such as non-representativeness of sample, study design, important uncontrolled confounders. | See discussion section on strengths and limitations. |
| Interpretations | 15 | Give a cautious overall interpretation of results, based on potential biases and imprecisions and suggest areas for future research. | See discussion and conclusion. Main objective of study is descriptive. |
| Generalizability | 16 | Discuss the external validity of the results. | External validity is not explicitly discussed. Since the main objective of this study is descriptive, it is mostly applicable to the researchers, peer-reviewers and journal editors with the sample characteristics described in the first paragraph of the results. |
| **Other sections** | | |  |
| Role of funding source | 17 | State whether any funding organization has had any roles in the survey’s design, implementation, and analysis. | See declarations section: Funding. There was no funding for this research project. |
| Conflict of interest | 18 | Declare any potential conflict of interest. | See declarations section: Competing interests. None were declared by authors. |
| Acknowledgements | 19 | Provide names of organizations/persons that are acknowledged along with their contribution to the research. | See declarations section: Acknowledgements. |
