# Supplementary material for: Perspectives on systematic review protocol registration: a survey amongst stakeholders in the clinical research publication process
Source: Syst Rev. 2023 Dec 14;12:234. doi: 10.1186/s13643-023-02405-z (PMC10720136; doi:10.1186/s13643-023-02405-z)
Supplement: Supplementary file 1 — Additional file 1. Schematic flow of questions and sections of the survey for (A) researchers and peer reviewers and (B) journal editors. [file 13643_2023_2405_MOESM1_ESM.pdf]

Additional file 1. Schematic flow of questions and sections of the survey for (A) researchers and peer reviewers, and (B) journal editors

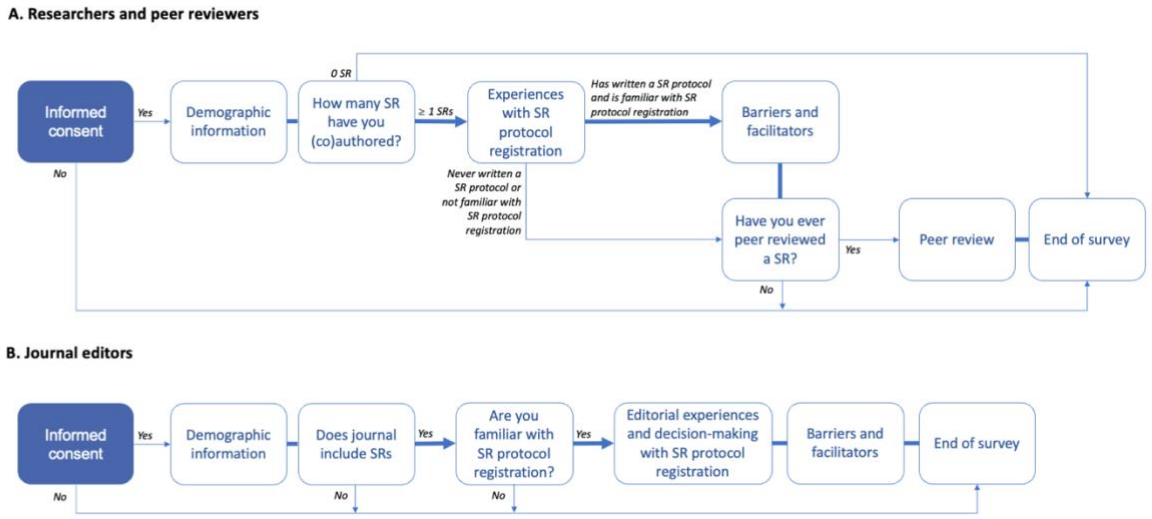


SR: systematic review
